# Relationship Between Oral Hypofunction and Nutritional Status in Patients Treated With Maxillofacial Prostheses

**DOI:** 10.7759/cureus.69558

**Published:** 2024-09-16

**Authors:** Michiko Isomura, Fumi Yoshioka, Shogo Ozawa, Jun Takebe

**Affiliations:** 1 Department of Removable Prosthodontics, School of Dentistry, Aichi Gakuin University, Nagoya, JPN

**Keywords:** cleft lip & palate, head and neck tumors and diseases, maxillofacial prosthesis, oral function, tongue defect

## Abstract

Introduction

Patients who have jaw or tongue defects owing to head and neck cancer or congenital disease suffer from oral dysfunction such as speech, swallowing, and mastication. Although maxillofacial prostheses are applied to improve oral functions, their oral functions remain compromised compared to those of patients without jaw or tongue defects. On the other hand, it has been reported that oral hypofunction could lead to malnutrition in the general elderly. However, to date, no reports have evaluated the relationship between oral function and nutritional status in patients with maxillofacial prostheses. In this study, we comprehensively evaluated oral function according to the examination items for oral hypofunction. We assessed which functions were related to nutritional status in patients with jaw defects caused by tumors or congenital diseases who are candidates for maxillofacial prosthetic treatment.

Methods

Patients who underwent prosthetic treatment at the Department of Maxillofacial Prosthodontics, Aichi-Gakuin University Dental Hospital, between May 2021 and January 2024 and who were doing well were included in the study. Oral function was assessed with seven tests according to the criteria of the Japanese Society of Geriatric Dentistry, and nutritional status was evaluated using the Mini Nutritional Assessment Short-Form (MNA-SF). Patients were classified according to age and type of deficiency. Additionally, the presence or absence of oral hypofunction and malnutrition or risk of malnutrition were assessed. The impact of oral hypofunction tests and defect status on the risk of malnutrition was evaluated using a logistic regression model.

Results

Oral hypofunction and malnutrition or risk of malnutrition were more frequent in the elderly group by age. Regarding the type of defect, the rate of oral hypofunction and malnutrition or risk of malnutrition was highest in the tongue defect group. Results of the logistic regression analysis indicated that nutritional status was associated with tongue deficiency, tongue-lip motor function, tongue pressure, and swallowing function.

Conclusion

The nutritional status of maxillofacial prosthetic wearers was associated with oral hypofunction. Particularly, malnutrition and risk of malnutrition were increased with tongue defects and deficiencies in tongue-lip motor function, tongue pressure, and swallowing.

## Introduction

The incidence of oral cavity cancer in Japan has recently increased [1]. The surgical treatment of oral cavity cancer often involves the removal of the jawbone and surrounding soft tissues, resulting in defects in the maxillofacial region and various functional and aesthetic disturbances. The postoperative use of a maxillofacial prosthesis can improve masticatory, swallowing, and articulatory function, and, thereby, improve oral health-related quality of life (OHRQOL) [2]. Particularly, swallowing function can be improved by wearing an obturator [3], while oral dysfunction caused by defects in the maxillofacial region, such as in articulation, mastication, and swallowing, can be improved by a maxillofacial prosthesis [4,5].

On the other hand, congenital maxillary defect owing to cleft lip and palate (CLP) has a standard treatment protocol including plastic surgery, oral surgery, and orthodontic treatment [6]. However, clinicians sometimes face patients with jaw defects who deviate from the standard treatment protocol or grow up without the opportunity for the treatment. Maxillofacial prostheses could be applied to these jaw defects.

Among studies on the relationship between masticatory function and frailty in the elderly, Iwasaki et al. reported that people with poor oral health prefer foods with low nutritional value [7,8]. It is known that oral hypofunction leads to poor physical function, and also to feeding and swallowing difficulties, which in turn lead to malnutrition [9,10]. However, to date, no studies have examined the association between oral function and nutritional status of maxillofacial prostheses wearers.

Here, we investigated the relationship between oral function and nutritional status in patients with maxillofacial prostheses using a simple nutritional status evaluation method and evaluated oral function in these patients using tests for oral hypofunction.

This article was previously presented as a meeting abstract at the 2023 AAMP Annual Conference on October 21-24, 2023.

## Materials and methods

Participants

Patients who visited the Maxillofacial Prosthetic Clinic of Aichi-Gakuin University Dental Hospital between May 2021 and January 2024 were recruited. Eligibility was restricted to patients with jaw defects because of tumors or congenital diseases who had undergone prosthetic treatment and had achieved good progress subjectively more than three months after delivery of prostheses. Informed consent was obtained before the study. We excluded patients with soft palate defects only, those with fixed prosthetic appliances, and those with communication difficulties. As this was a case-control retrospective study, no a priori sample size calculation was required. The study was approved by the Ethics Committee of Aichi-Gakuin University (Approval No. 632).

Measurement

We first assessed the nutritional status of patients using the Mini Nutritional Assessment Short-Form (MNA-SF) questionnaire and their swallowing function using the Eating Assessment Tool (EAT-10) questionnaire [11,12]. We then examined the following six tests of oral hypofunction.

Seven tests of oral hypofunction (i.e., oral hygiene, oral dryness, occlusal force, tongue pressure, tongue and lip function, masticatory function, and swallowing function) were examined according to the standards of the Japanese Society of Geriatric Dentistry. Patients with scores below the standard values for three or more of the seven tests were defined as having oral hypofunction [13]. The examination was performed with the patient wearing their prosthesis. In patients who experienced patient fatigue or found it difficult to perform the seven tests, only those tests that could be performed were measured.

Oral Hygiene

A sterile cotton swab was attached to a constant-pressure specimen collection device and rubbed three times around the central 1 cm area of the dorsum of the tongue. If the tongue was removed, the sample was taken from the flap. The total microbial count of the specimen was then determined using a bacteria counter (Bacteria counter, Panasonic Healthcare Co, Ltd, Japan). The reference value was 6.5 log10 (colony-forming unit (CFU)/mL)(Level 4) or higher, indicating poor oral hygiene.

Oral Dryness

A 1 cm area at the tip of the dorsum of the tongue was measured using an oral moisture checker (Mucus®, Life Co, Ltd, Japan). If measurement was difficult, this was because of the tongue, which included a reconstructed flap the buccal mucosa that was used for measurement. Values below the standard value of 27.0 were considered to indicate oral dryness.

Occlusal Force

Patients were then asked to place their heads firmly on the headrest of the chair. Pressure-indicating film (Dental Prescale II, GC Co Ltd., Japan) was aligned with the dentition, and the subject was instructed to bite down for three seconds. Microcapsules built into the film were thereby broken, and the resulting colored areas were converted into bite force using the attached bite force analyzer. Three measurements were taken with the prosthesis in place, and the average value was used. In these measurements, a decrease in occlusal force was diagnosed for values below the standard value of 350 N as automatic cleaning was conducted using the Prescale II pressure filter function.

Tongue Pressure

After connecting the tongue pressure probe to a tongue pressure measuring instrument (TPM-01, JMS Co, Ltd, Japan) and adjusting the internal pressure, the patient was asked to insert the tongue pressure probe into the mouth, grasp the hard part of the probe with the front teeth, and keep pressing the balloon against the palate with the tongue for seven seconds. The balloon portion of the probe tends to shift when pressed against the tongue, so measurements were taken with care. After practicing once, measurements were taken three times and the average value was used. Low tongue pressure was defined as a value below a reference value of 30 kilopascals (kPa).

Tongue-Lip Motor Function

The patient was asked to pronounce the syllables /Pa/, /Ta/, and /Ka/ repeatedly for five seconds. Each syllable was pronounced and measured independently from the other two. The number of syllables pronounced per second was determined using an automatic counter (Kenkokun Handy, Takei Scientific Instruments Co, Ltd, Japan). A value below a standard value of six times per second for any of /Pa/, /Ta/, or /Ka/ indicates a decrease in tongue-lip motor function.

Masticatory Function

Glucose-containing gummies were masticated for 20 seconds and then the mouth was rinsed with 10 mL of water. The chewed gummi and water were spat into a funnel. The filtered solution was collected with a disposable collection brush, and placed on a sensor chip, and the amount of eluted glucose was then measured using a masticatory ability testing system (Gluco Sensor GS-2, GC Co. Ltd., Japan). Decreased masticatory ability was defined as a glucose elution level of less than 100 mg/dL.

Swallowing Function

EAT-10 consists of 10 questions about swallowing. Each question is rated on a scale of 0-4, where 0 indicates no problem and scores 1-4 indicate increasing degrees of a problem. A total score of 3 or higher indicates a swallowing problem [12].

Nutrition Status

Nutritional status was assessed using the MNA-SF. The MNA-SF consists of measurements of weight and height, as well as questions about a decrease in food intake over the past three months, the ability to walk on one's own, and the presence of acute illness, stress, or neuropsychiatric disorders. Each question is scored on a scale of 0-2 or 0-3. A total score of less than 8 points is considered "malnutrition," 8-11 points are "at risk of malnutrition," and 12 or more points are "normal nutrition" [11].

Statistical Analysis

The impact of oral hypofunction tests and defect status on the risk of malnutrition was evaluated using a logistic regression model. Defect status was classified into five deficiency groups, namely, a maxillary defect group, a mandibular defect group, a maxillomandibular defect group, a tongue defect group, and a CLP group. Subjects who had both mandible and tongue defects were included in the mandibular defect group. Odds ratios (ORs) and corresponding 95% CIs for continuous or categorical variables of oral functional and demographic status were estimated. All statistical analyses were performed using STATA version 16 (StataCorp LLC, College Station, TX). All tests were two-sided, and P values<0.05.

## Results

**Figure 1:**
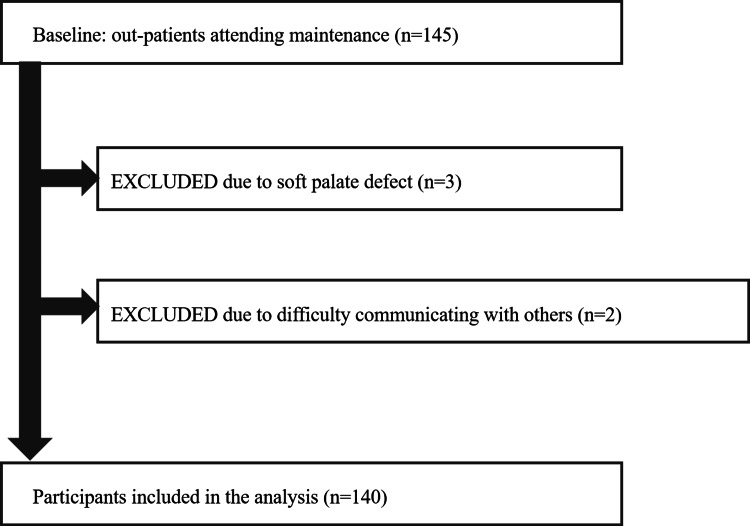
Flow chart of participants

One hundred and forty-five patients who had undergone maxillofacial prosthetic treatment at the Maxillofacial Prosthetic Clinic, Aichi-Gakuin University Dental Hospital, between May 2021 and January 2024 were identified. Figure 1 shows the selection criteria.

Patients were 74 males and 66 females with a mean age of 67.9 ± 13.3 years. Of these, 89 patients aged 65 years or older, with a mean age of 76.1 ± 5.7 years, were grouped as elderly, and 51 patients aged younger than 65 years, with a mean age of 53.5 ± 10.0 years, were grouped as young.

Defect status was as follows: 69 patients had maxillary defects, 37 had mandibular defects, 11 had maxillomandibular defects, 11 had tongue defects, and 12 had CLP. As prosthetic devices, the patients were provided with maxillary prostheses for maxillary defects, mandibular prostheses and occlusal ramps for mandibular defects, maxillomandibular prostheses and occlusal ramps for maxillomandibular defects, palatal augmentation prostheses (PAP) auxiliaries for tongue defects, and jaw prostheses for CLP.

**Figure 2:**
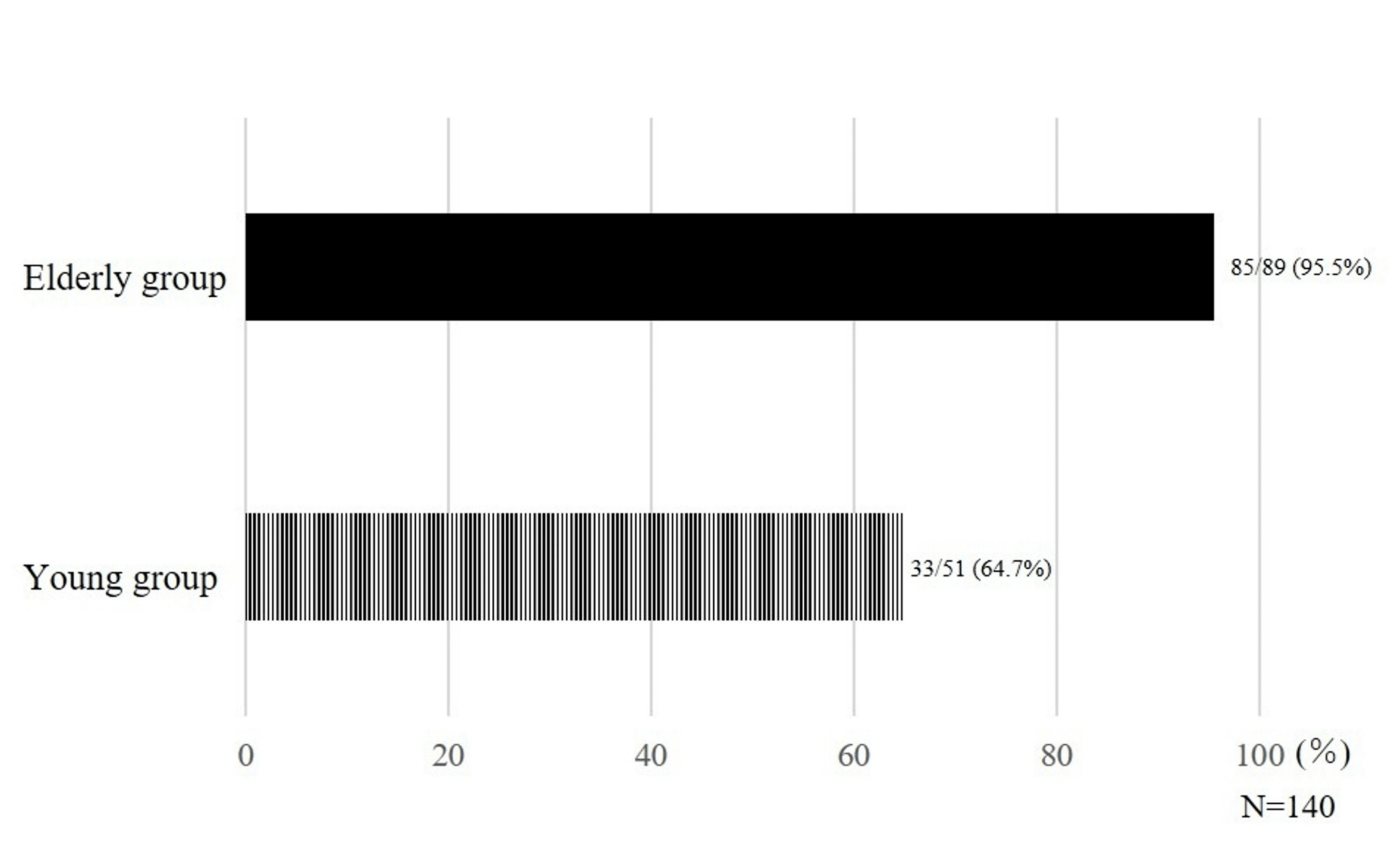
Ratio of oral hypofunction by age The elderly group had a higher rate of oral hypofunction compared to the young group.

Patients who showed oral hypofunction were classified by age. Results showed that 85 out of 89 patients (95.5%) in the elderly group had oral hypofunction compared with 33 out of 51 patients (64.7%) in the young group (Figure 2).

**Figure 3:**
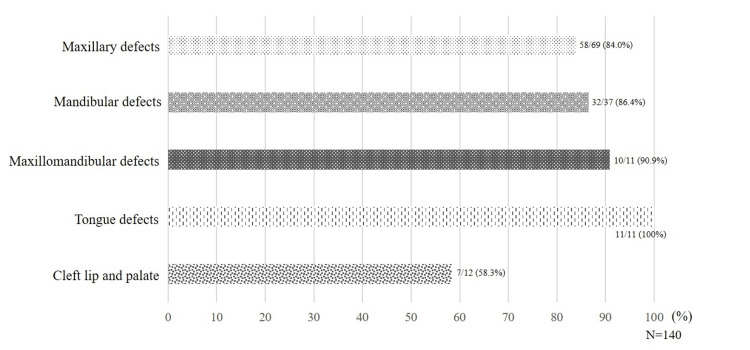
Ratio of oral hypofunction by type of defect All patients in the tongue-deficient group had oral hypofunction.

On classification by state of defect, oral hypofunction was observed in 58 out of 69 patients (84%) in the maxillary defects group, 32 out of 37 patients (86.4%) in the mandibular defects group, 10 out of 11 patients (90.9%) in the maxillomandibular defects group, 11 out of 11 patients (100%) in the tongue defects group, and seven out of 12 patients (58.3%) in the CLP group (Figure 3).

**Figure 4:**
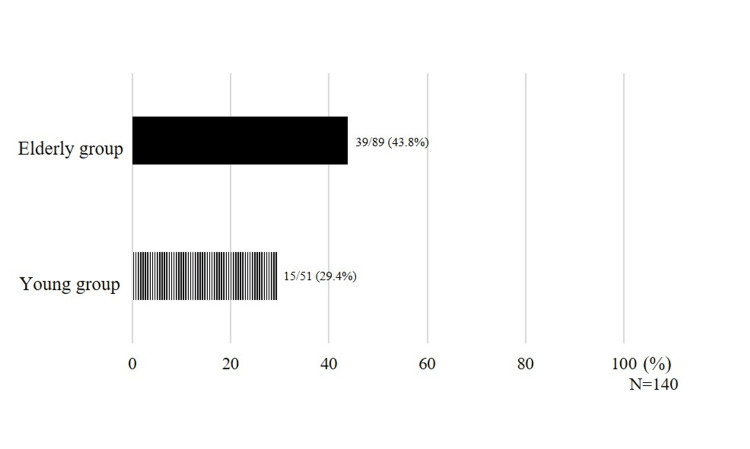
Ratio of malnutrition and risk of malnutrition by age The ratio of patients with malnutrition or at risk of malnutrition was higher in the elderly group.

Patients who showed malnutrition or risk of malnutrition were classified by age. The results showed that 39 out of 89 patients (43.8%) in the elderly group had malnutrition or risk of malnutrition compared with 15 out of 51 (29.4%) in the young group (Figure 4).

**Figure 5:**
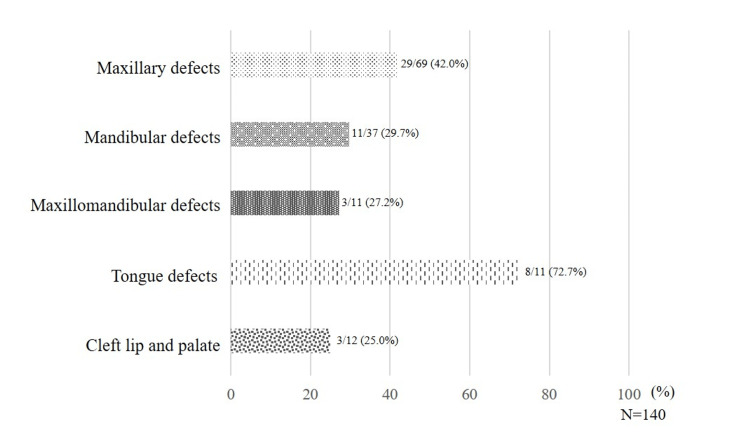
Ratio of malnutrition and risk of malnutrition by the state of the defect The ratio of patients with malnutrition or at risk of malnutrition was the highest in the tongue defect group.

On classification by state of defect, malnutrition, or risk of malnutrition was seen in 29 out of 69 patients (42.0%) in the maxillary defects group, 11 out of 37 patients (29.7%) in the mandibular defects group, three out of 11 patients (27.2%) in the maxillomandibular defects group, eight out of 11 patients (72.7%) in the tongue defect group, and three out of 12 patients (25%) in the CLP group (Figure 5).

The results of logistic regression analysis are shown in Table 1.

**Table 1:** The impact of oral hypofunction tests and defect status on the risk of malnutrition Abbreviation: OR, odds ratio; CI, confidence interval; CLP, cleft lip and palate; *continuous variable; Significant differences were found in tongue defects, tongue-lip function, tongue pressure, and swallowing function.

Variables		N	OR	95%Cl	P value
Sex					
	Male	74	1.00	Reference	-
	Female	66	0.54	0.27-1.08	-
Age					
	≥65	89	0.83	0.22-3.18	0.781
	<65	51	0.49	0.14-1.65	0.249
Defect					
	Mandile	37	1.00	Reference	-
	Maxillomandibular	11	1.13	0.25-5.07	0.875
	CLP	12	1.27	0.29-5.60	0.753
	Tongue	11	0.16	0.04-0.71	0.016
	Maxillary	69	0.62	0.26-1.45	0.271
Oral Hygiene					
	1	17	1.00	Reference	-
	2	32	1.04	0.30-3.58	0.949
	3	44	0.87	0.27-2.78	0.809
	4	33	0.95	0.28-3.24	0.941
	5	14	0.55	0.13-2.31	0.411
Oral Dryness*			0.97	0.85-1.11	0.681
Occlusal Force*			1.00	0.999-1.001	0.462
Tongue Pressure*			1.05	1.02-1.09	0.003
Tongue-Lip Motor Function*					
	Pa		1.34	1.06-1.68	0.013
	Ta		1.48	1.17-1.87	0.001
	Ka		1.59	1.21-2.07	0.001
Masticatory Function*			1.00	0.995-1.007	0.648
Swallowing Function*			0.29	0.14-0.59	0.001

Significant differences were seen in the tongue deficiency group, and for tongue-lip motor function, tongue pressure, and swallowing function.

## Discussion

In this study of patients with jaw defects because of tumors or congenital diseases who had undergone treatment with maxillofacial prostheses, we found that oral hypofunction and malnutrition or risk of malnutrition were more frequent in the elderly group by age. Particularly, nutritional status was associated with tongue deficiency, tongue-lip motor function, tongue pressure, and swallowing function. Conversely, no association was observed between nutrition status and oral hygiene, oral dryness, occlusal force, and masticatory function in these patients. To our knowledge, this is the first study to investigate the relationship between oral function and nutritional status in patients treated with maxillofacial prostheses.

When oral defects occur congenitally or after surgery for malignant tumors, or when the morphology of the oral cavity changes because of reconstruction such as with flaps, the range of motion is restricted. This in turn results in functional impairment, such as in feeding, swallowing, and speech. Any patient with a defect treated with a prosthesis or flap should be evaluated for nutritional status to ensure that quality of life is maintained. Understanding the degree of functional disability and nutritional status is critical for future rehabilitation and quality of life.

In this study, we found that the ratio of oral hypofunction by age was higher in the elderly group than in the young group. A previous study found that the rate of oral hypofunction in patients who visited a dental hospital aged 65 years or older was 35.9% [14], while another reported a rate of oral hypofunction in patients with gastric cancer of 25.3% [15]. The rate of oral hypofunction in our elderly group was much higher than in these reports. In addition to changes because of aging, our elderly group may have first presented with a decreased number of remaining teeth, lack of bony support, and weakened oral myofunction because of postoperative sequelae.

All patients in the tongue defect group showed oral hypofunction. Generally, the greater the extent of the tongue defect, the more restricted tongue movement is because of the reduced volume of the tongue. In patients with mandibular and tongue defects, previous reports have noted that the excision of the external lingual muscle and hypoglossal nerve affects the movement of the tongue. Tongue function plays a role in the items examined to test for oral hypofunction, including tongue lip motor function, tongue pressure, and swallowing function, and it is, therefore, reasonable that all subjects in the tongue defect group had oral hypofunction.

As with nutritional status, the elderly group was also more likely than the young group to either have malnutrition or be at risk of malnutrition. Previous studies reported that 2.4% of the general elderly population aged 65-74 had malnutrition and that 39.5% were at risk of malnutrition. Respective percentages for the elderly population aged 75-84 years were 10.2% and 48.7% [16]. In our present study, 43.8% of the elderly group and 29.4% of the young group either had malnutrition or were at risk for malnutrition. We speculate that in addition to the general decline in physical condition after cancer surgery, the feeding-related function also deteriorates as a consequence of each oral defect.

By the type of defect, the tongue defect group was the most likely to have either malnutrition or be at risk of malnutrition. We consider that most patients in the tongue defect group had functional insufficiency of the suprahyoid and sublingual muscle groups and consequent difficulty in ingestion and swallowing. Additionally, tongue defects reduce the volume of the tongue and restrict tongue movement, which in turn decreases the ability of the tongue to form a food bolus and deliver it to the pharynx. These feeding and swallowing difficulties result in either malnutrition or risk of malnutrition. Logistic regression analysis revealed significant associations between nutrition status and tongue and lip motor function, tongue pressure, swallowing function, and the tongue defect group. Conversely, no significant relations were found between nutritional status and masticatory function. Tongue-lip motor function, tongue pressure, and swallowing function are all closely associated with food bolus formation and the ability to deliver the bolus to the pharynx during deglutition [17].

As these functions deteriorate, it becomes necessary to devise new forms of food, such as soft foods and foods with a thickening effect. About nutrition, protein and vitamin intake are generally important for good nutrition. Proteins and vitamins are muscle-related nutrients, and their intake is associated with oral hypofunction in the elderly [18-21]. Foods high in protein and vitamins, such as meat, fish, and vegetables, are often hard and fibrous and, therefore, difficult for elderly people with oral hypofunction and those with trouble ingesting and swallowing to consume. Moreover, patients with tongue defects tend to avoid these foods and instead consume foods with high carbohydrate and calorie contents, such as rice, which are soft and easy to eat. As a result, protein and vitamins are not consciously consumed, and the intake of foods rich in them tends to be insufficient [22-24]. These patients could therefore either have malnutrition or be at risk of malnutrition.

In this study, the masticatory function was not directly related to malnutrition or the risk of malnutrition. Even if the masticatory function of elderly patients with dentures improves, their nutritional status does not improve unless the patient's nutritional awareness is changed through nutritional guidance intervention [25-29]. We saw a similar trend in patients with maxillofacial prostheses in this study. Although patients with a jaw defect had acquired some manner of occlusal contact through maxillary or mandibular prostheses, regaining normal function in the surrounding soft tissue such as the tongue, buccal mucosa, lip or soft palate, whose coordination with occlusion is necessary for feeding, was difficult. It is therefore important to provide patients wearing maxillofacial prostheses with dietary guidance from a dietitian, in collaboration with multiple other disciplines, even after the maxillofacial prosthesis is first provided.

Several limitations of our study warrant mention. First, evaluation of oral function was difficult. The oral function tests in this study were primarily designed to test for oral hypofunction in the general population of the elderly. Some tests, such as tongue pressure and tongue lip motor function, include actions that are technically difficult for maxillofacial-deficient individuals to perform. Tongue pressure measurement is particularly difficult for patients with trismus as it is difficult to insert the probe and hold it in the correct position while pressed against the palate. In patients with reconstruction with a flap on the tongue or palate, the probe may not even be pressed against the palate owing to its flexibility. In such cases, tongue pressure was measured after several additional practice sessions to the number in other subjects, until the patient became used to holding and stably pressing the probe. The tests of tongue and lip motor function require the subject to pronounce /pa/, /ta/, and/ka/ repeatedly for five seconds. Some patients had decreased lung capacity owing to postoperative health conditions or age, which hindered recognition by the testing instrument although pronunciation occurred. Second, the location of the primary site was heterogeneous, and we, therefore, accumulated patients other than those with maxillary defects for future investigation. Additionally, although the MNA-SF was used to assess the nutritional status of patients, a more detailed analysis using the brief-type self-administered diet history questionnaire (BDHQ) is necessary: this will reveal which nutrients are related to malnutritional status and oral function and thereby allow more detailed dietary guidance [30].

To our knowledge, this is the first study to investigate the relationship between oral function and nutritional status in patients treated with maxillofacial prostheses. Future studies should examine the association of social support and nutritional status on the quality of life in these patients.

## Conclusions

In this study, we found that oral hypofunction in wearers of maxillofacial prosthetics was associated with nutritional status. Particularly, tongue defects, decreased tongue-lip function, low tongue pressure, and swallowing dysfunction were associated with an increased risk of malnutrition and malnutrition. These results could contribute to establishing the clinical management of nutritional support for patients with jaw or tongue defects. A future study including an analysis of detailed nutrients will support the healthcare for those patients.
